# Recurrent lower gastrointestinal bleeding from idiopathic ileocolonic varices: a case report

**DOI:** 10.1186/1752-1947-4-257

**Published:** 2010-08-10

**Authors:** Ravula Phani Krishna, Rajneesh Kumar Singh, Uday C Ghoshal

**Affiliations:** 1 Department of Surgical Gastroenterology, Sanjay Gandhi Post-graduate Institute of Medical Sciences, Lucknow 226014, India; 2 Department of Medical Gastroenterology, Sanjay Gandhi Post-graduate Institute of Medical Sciences, Lucknow 226014, India

## Abstract

**Introduction:**

Varices of the colon are a rare cause of lower gastrointestinal bleeding, usually associated with portal hypertension due to liver cirrhosis or other causes of portal venous obstruction. Idiopathic colonic varices are extremely rare. Recognition of this condition is important as idiopathic colonic varices may be a cause of recurrent lower gastrointestinal bleeding.

**Case presentation:**

We report the case of a 21-year-old Asian man from north India who presented with recurrent episodes of lower gastrointestinal bleeding. Colonoscopy revealed varices involving the terminal ileum and colon to the sigmoid. Thorough evaluation was undertaken to rule out any underlying portal hypertension. Our patient underwent subtotal colectomy including resection of involved terminal ileum and an ileorectal anastomosis.

**Conclusion:**

Colonic varices are an uncommon cause of lower gastrointestinal bleeding. Idiopathic colonic varices are diagnosed after excluding underlying liver disease and portal hypertension. Recognition of this condition is important as prognosis is good in the absence of liver disease and is curable by resection of the involved bowel.

## Introduction

Varices of the colon are a rare cause of lower gastrointestinal bleed, usually associated with portal hypertension due to liver cirrhosis or other causes of portal venous obstruction. Idiopathic colonic varices are extremely rare. Recognition of this condition is important as idiopathic colonic varices may be a cause of recurrent lower gastrointestinal bleed.

## Case report

**Figure 1:**
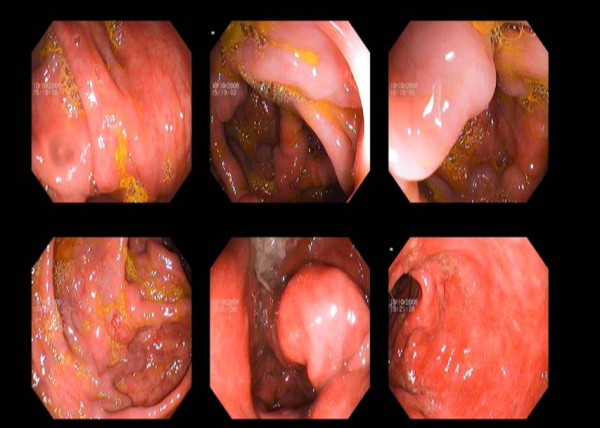
**Colonoscopy shows dilated tortuous veins extending throughout the colon**.

**Figure 2:**
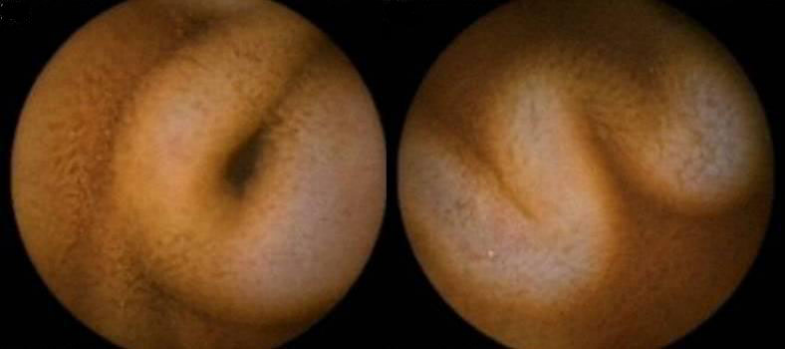
**Capsule endoscopy shows dilated tortuous veins in terminal ileum**.

A 21-year-old Asian man from north India presented with history of recurrent episodes of lower gastrointestinal bleeding over the past six years. He had intermittent episodes, one to two per year, of passing bloody maroonish stools with occasional hematochezia. Episodes were self limited lasting two to three days, but required repeated hospital admissions with multiple blood transfusions. He had no significant past medical or family history of similar complaints. Physical examination was unremarkable. He was admitted with a fresh episode of lower gastrointestinal bleeding, which subsided spontaneously. Coagulation profile, liver function tests and hepatitis serology were normal. Upper gastrointestinal endoscopy to the third part of the duodenum did not reveal findings suggestive of portal hypertension or any other bleeding source. Colonoscopy revealed several large dilated tortuous sub-mucosal varices extending from the upper rectum, sigmoid, the entire colon extending into the terminal ileum (Figure 1). Our patient was examined for portal hypertension. Doppler ultrasound revealed normal liver size and echotexture, portal vein 10 mm, splenic vein 7 mm with normal hepatopetal flow and no evidence of collaterals. Magnetic resonance portovenogram revealed no evidence of cirrhosis or portal hypertension. Selective mesenteric angiography was carried out to search for any other vascular lesions in the gastrointestinal tract. All vascular territories were found to be normal and colonic lesions were undetected on an angiogram. Small bowel evaluation with enteroclysis was normal. However, capsule enteroscopy revealed evidence of tortuous dilated vessels in distal ileum (Figure 2).

**Figure 3:**
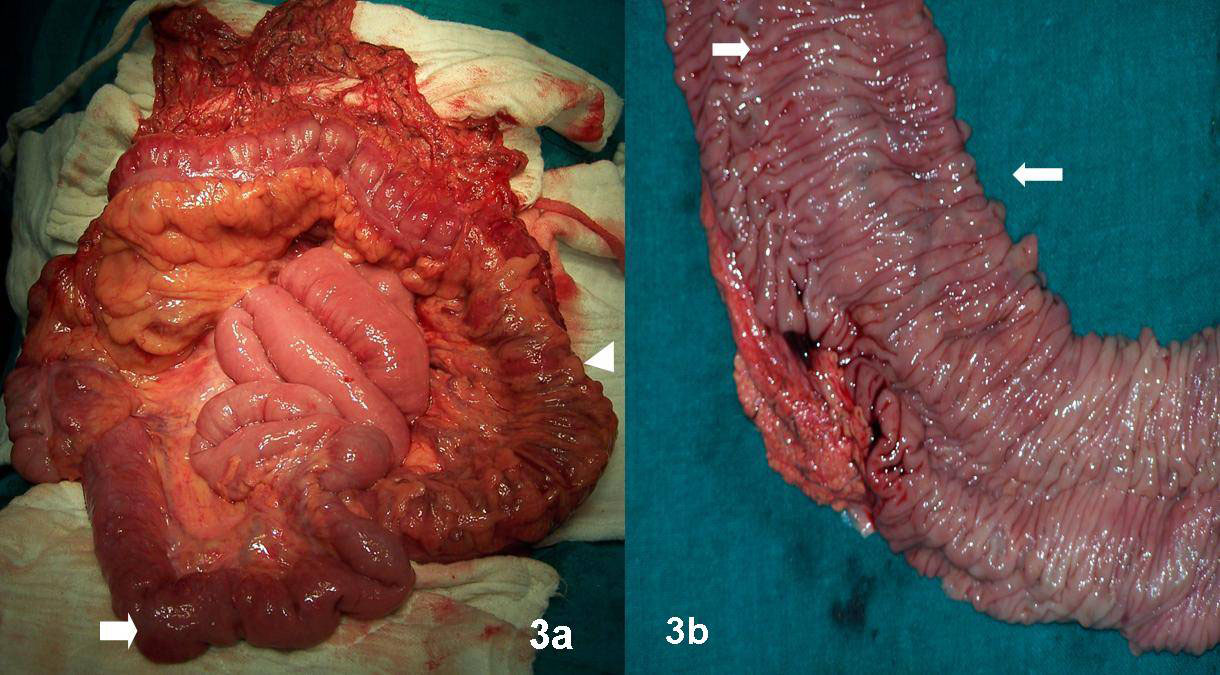
**(A) Terminal ileum shows evidence of tortuous dilated veins on surface (arrows) with clear demarcation from proximal small bowel**. Colon normal except for few dilated veins over surface of sigmoid (arrowhead). (B) Cut specimen of colon shows prominent tortuous sub-mucosal vessels (arrows).

At surgery his liver was normal and there was no evidence of portal hypertension. Intra-operatively, portal pressure measured by cannulating mesenteric veins was normal. Small bowel was normal except for the terminal 15 cm, which showed evidence of dilated tortuous sub-serosal vessels with a clear demarcation from rest of small bowel marked by a meandering dilated mesenteric vein (Figure 3a), confirmed by intra-operative enteroscopy. Serosal aspect of colon was normal except few dilated veins at sigmoid (Figure 3a). There were no collaterals in the colonic mesentery or retroperitoneum. Sub-total colectomy including the terminal ileum was performed with an ileorectal anastomosis. The rectum was relatively spared of varices. The colectomy specimen revealed multiple dilated tortuous sub-mucosal vessels in the colon (Figure 3b). Histology revealed large dilated thin walled vascular channels in the submucosa.

## Discussion

Lower gastrointestinal bleeding is a frequent cause of hospital admissions. Common causes of lower gastrointestinal bleeding are diverticulae, vascular ectasia, colitis, non-specific caecal ulcers, neoplasia and proctal lesions [1]. Varices of the colon are a rare cause of lower gastrointestinal bleed. Incidence in one autopsy series was 0.007% [2]. Varices are usually associated with portal hypertension with the most common locations being the rectosigmoid and ceacum. In one study among cirrhotics, colonic varices were present in 31%, but bleeding from colonic varices was seen in only 1% [3]. Other less common causes of colonic varices are congestive heart failure, mesenteric vein thrombosis, pancreatitis with splenic vein thrombosis and post-operative adhesions [1]. Idiopathic varices are rare. Establishing a diagnosis of idiopathic colonic varices needs exclusion of other etiologies such as cirrhosis and portal venous obstruction by thorough evaluation. Several reported cases have shown an associated familial aggregation. To date only 10 cases of familial colonic varices and an additional 10 cases with no other family members affected have been reported [4-11]. Most patients present before their third decade. This strongly suggests that these varices may be congenital and represent an inbred vascular anomaly. In patients with familial involvement the numbers are too small to draw any conclusion on possible modes of inheritance. Some authors have suggested a possible autosomal recessive mode of inheritance [9]. Even in patients with late presentation, after their fifties, it is unlikely to be vascular degenerative ectasia. It is important to note that even in cirrhotics and non-cirrhotic patients it is unusual for varices to extend beyond the anorectal area, in contrast with these patients. In approximately half the cases the whole colon is involved and in cases with segmental lesions right and left colon are equally involved [5]. Thus it is probably more likely that all these cases represent a significant inborn vascular anomaly [9].

Usual presentation is recurrent massive bleed. The age at presentation has ranged from 18 years to 75 years with no sexual predilection [4-9]. Bleeding is usually painless, but may occasionally be associated with crampy abdominal pain. Colonoscopy is the investigation of choice and varices can be visualized as dilated tortuous venous channels. However, varices may occasionally be mistaken for polyposis or tumor. Colonoscopy during a period of hypotension, along with compression of varices due to insufflation, may cause them to be missed [2]. Barium enema is unreliable and varices may be missed or mistaken for polyps [5]. Mesenteric angiography is a useful diagnostic tool, but diagnosis of colonic varices may be missed on angiography as seen in our case and other case reports [8,10]. Varices are detected in the venous phase of angiography and the volume of contrast used may not be sufficient to demonstrate varices [8]. Before concluding that true idiopathic varices are present, underlying cirrhosis and portal venous obstruction should be excluded. Liver function tests, hepatitis serology, Doppler studies for portal venous system, portovenogram and wedge hepatic venous pressure measurements should be done to rule out liver disease or portal venous obstruction. In patients with involvement of the entire colon, extension into the ileum has been reported [4], as seen in our case. Therefore these patients also need pre-operative evaluation of small bowel as well as careful intra-operative assessment by inspection and intra-operative endoscopy as needed to delineate extent of small bowel involvement.

Treatment options are conservative management and surgical resection. Conservative treatment alone has been attempted [3,9]; however, surgical resection of the involved colon is the treatment of choice in view of the risk of recurrent bleed [4-8,10]. Unlike patients with portal hypertension and hepatocellular disease, colonic resection can be performed with low morbidity and mortality in this group of patients [4,8]. For patients who are poor surgical candidates, due to advanced age or co-morbidities, a conservative approach may be justified [3,9]. Prognosis of idiopathic colonic varices is good at all ages compared with cirrhosis due to the low pressure within varices and absence of associated liver disease.

Our case highlights a rare cause of lower gastrointestinal bleed of which fewer than 20 cases have been reported in literature. Knowledge of this entity is important for the treating physicians as it is commonly confused with portal hypertensive varices. Identification of true idiopathic varices in such cases by a thorough workup has important prognostic and therapeutic implications.

## Conclusions

Colonic varices are an uncommon cause of lower gastrointestinal bleed. Idiopathic colonic varices are diagnosed after excluding underlying liver disease and portal hypertension. Family history of similar problem may be seen in some, but not all, cases. Recognition of this condition is important as prognosis is good in the absence of liver disease and is curable by resection of involved bowel.

## Consent

Written informed consent was obtained from the patient for publication of this case report and any accompanying images. A copy of the written consent is available for review by the journal's Editor-in-Chief.

## Competing interests

The authors declare that they have no competing interests.

## Authors' contributions

RPK and RKS were involved in conceiving the study and drafting the manuscript. UCG was involved in revision of draft. All authors were involved the management of the patient, contributed intellectual content and have read and approved the final manuscript.
